# Physical activity before radical prostatectomy reduces sick leave after surgery - results from a prospective, non-randomized controlled clinical trial (LAPPRO)

**DOI:** 10.1186/s12894-016-0168-0

**Published:** 2016-08-16

**Authors:** E. Angenete, U. Angerås, M. Börjesson, J. Ekelund, M. Gellerstedt, T. Thorsteinsdottir, G. Steineck, E. Haglind

**Affiliations:** 1 Department of Surgery, Institute of Clinical Sciences, Sahlgrenska Academy at University of Gothenburg, SSORG, Sahlgrenska University Hospital/Östra, SE-416 85 Gothenburg, Sweden; 2 University West, Trollhättan, Sweden; 3 Swedish School of Sport and Health Sciences, Stockholm, Sweden; 4 Department of Cardiology, Karolinska University Hospital, Stockholm, Sweden; 5 Faculty of Nursing, School of Health Sciences, University of Iceland, Reykjavik, Iceland; 6 Division of Clinical Cancer Epidemiology, Department of Oncology, Institute of Clinical Sciences, Sahlgrenska Academy at the University of Gothenburg, Gothenburg, Sweden; 7 Department of Oncology and Pathology, Division of Clinical Cancer Epidemiology, Karolinska Institutet, Solna, Sweden

**Keywords:** Prostatic neoplasm, Prostatectomy, Physical fitness

## Abstract

**Background:**

Studies have reported that early physical rehabilitation after surgical procedures is associated with improved outcome measured as shorter hospital stay and enhanced recovery. The aim of this study was to explore the relationship between the preoperative physical activity level and subsequent postoperative complications, sick-leave and hospital stay after radical prostatectomy for prostate cancer in the setting of the LAPPRO trial (LAParoscopic Prostatectomy Robot Open).

**Methods:**

LAPPRO is a prospective controlled trial, comparing robot-assisted laparoscopic and open surgery for localized prostate cancer between 2008 and 2011. 1569 patients aged 64 or less with an occupation were included in this sub-study. The Gleason score was <7 in 52 % of the patients. Demographics and the level of self-assessed preoperative physical activity, length of hospital stay, complications, quality of life, recovery and sick-leave were extracted from clinical record forms and questionnaires. Multivariable logistic regression, with log-link and logit-link functions, was used to adjust for potential confounding variables.

**Results:**

The patients were divided into four groups based on their level of activity. As the group with lowest engagement of physical activity was found to be significantly different in base line characteristics from the other groups they were excluded from further analysis. Among patients that were physically active preoperativelly (*n* = 1467) there was no significant difference between the physical activity-groups regarding hospital stay, recovery or complications. However, in the group with the highest self-assessed level of physical activity, 5-7 times per week, 13 % required no sick leave, compared to 6.3 % in the group with a physical activity level of 1-2 times per week only (*p* < 0.0001).

**Conclusions:**

In our study of med operated with radical prostatectomy, a high level of physical activity preoperatively was associated with reduced need for sick leave after radical prostatectomy compared to men with lower physical activity.

**Trial registration:**

The trial is registered at the ISCRTN register. ISRCTN06393679.

## Background

Physical activity has gained increasing focus, as a life-style factor of importance with a number of studies have confirming its positive effects on cardiovascular and overall health [1]. It has been shown that self-assessed physical activity concurs well with the actual physical fitness of the individual [2].

In regard to cancer, physical activity may reduce the risk of cancer development, as shown by a review summarizing nearly 170 studies stating that the scientific evidence for the association between lack of physical activity and the development of cancer is convincing for breast and colon cancer and probably also for prostate cancer [3]. A high level of physical activity has also recently been reported to reduce overall and prostate-specific mortality in patients diagnosed with prostate cancer [4].

For postoperative rehabilitation, the benefits from preoperative physical activity in addition to a postoperative early rehabilitation schedule have been reported for spinal surgery [5] and is suggested for several types of cancer surgery [6], including prostate cancer [7]. In colorectal surgery the benefits of enhanced recovery programs, including early postoperative mobilization [8], have been clearly demonstrated. The impact of preoperative prehabilitation has been evaluated showing an improved cardio-pulmonary function [9, 10], however most studies have not used clinically important outcome measures such as complications, postoperative morbidity or length of hospital stay, although studies are underway and there are several indications that prehabilitation reduces hospital stay [11] [12].

Localized prostate cancer can be treated with radical prostatectomy [13], which can be performed either by open, laparoscopic or robot-assisted laparoscopic surgery [14, 15]. Radical prostatectomy is associated with a low overall operative morbidity and mortality and hospital stay is two days or less [15, 16], but there is still considerable long-term morbidity with urinary incontinence and decreased sexual health [17]. In recent years self-assessment evaluations on recovery have been developed in addition to measurements such as length of hospital stay [18, 19]. Another more precise measurement of recovery is time to return to work or total time of sick-leave. For patients operated with radical prostatectomy studies in the United States have indicated that 50 % were back to work after a two week period and to unrestricted activity within one month although these figures may vary between countries and between different types of surgery [20–22].

In the large prospective Swedish trial, LAPPRO (LAParoscopic Prostatectomy Robot Open), comparing robot-assisted laparoscopic radical prostatectomy to open radical prostatectomy as a definitive treatment for localized prostate cancer, patients answered detailed questionnaires on many aspects of quality of life including self-assessed physical activity and sick leave [23]. Short-term results have been published, indicating that robot-assisted laparoscopic prostatectomy is a safe procedure and that it has some advantages compared to open surgery, such as shorter hospital stay and less risk of reoperation during initial hospital stay [24] and long-term results show no statistical difference in urinary incontinence at one year, but somewhat less erectile dysfunction in the robot-assisted laparoscopically radical prostatectomy compared to open (70 % vs. 75 %) [25].

The aim of the present study was to determine the relationship between the patients’ self-assessed preoperative physical activity level and the postoperative course, including hospital stay and sick leave, after radical prostatectomy for localized prostate cancer in the setting of the LAPPRO trial.

## Methods

### Study design

The study-population derives from LAPPRO (LAParoscopic Prostatectomy Robot Open), a prospective, non-randomized controlled clinical trial comparing outcomes after open retropubic and robot-assisted laparoscopic radical prostatectomy [23]. Fourteen Swedish urological departments well established in performing radical prostatectomy included patients in the trial. Patient-reported data were collected before surgery as well as 3, 12 and 24 months after surgery. In addition, clinical record forms gathered information preoperatively, surgical data, follow-up data 6-12 weeks, 12 and 24 months after surgery. The study protocol has been published [23] and is available at www.ssorg.net.

### Patients

**Fig. 1:**
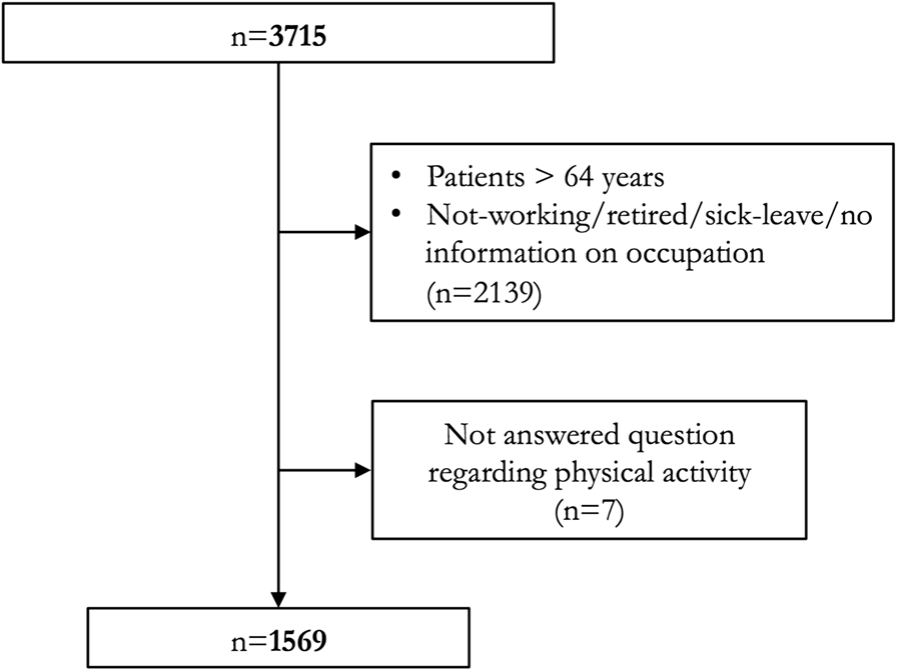
Flow-chart

A total of 3715 patients operated with a radical prostatectomy for clinically localized prostate cancer between September 2008 and November 2011 gave informed consent, and were included in the study (Fig. 1). For the pupose of this analysis, we focused on patients who could be in need of sick-leave. Thus we selected employed or un-employed patients, but did not include retired patients or those with full, chronic disability. To identify our target population out of the entire trial population we used results in questions asked in the baseline questionnaire, with the ensuing definitions based on answering categories. (*n* = 1576). Patients in the target population who did not answer the questionnaire in general or specifically the question regarding physical activity were excluded (*n* = 7). Left to analyze were 1569 patients, 397 patients were operated with open retropubic prostatectomy and 1170 patients were operated with robot-assisted laparoscopy, and two with an unidentified procedure. All patients were treated according to the local hospital routines, regarding preoperative and postoperative care. The patients were assigned a standardized period of short sick leave, with a possibility of additional sick leave by a simple phone call.

### Data collection

Patients were given questionnaires prior surgery in the outpatient clinic and at 3, 12 and 24 months. In this substudy only questionnaires prior surgery and at three months were analyzed. The questionnaires with patient-reported data were modified from previous studies [26, 27]. Questions concerning primary and secondary endpoints, possible confounders and effect modifiers were included and most questions had been used previously in other studies [28]. Qualitative open interviews with prostate cancer patients before and after surgery revealed new topics that resulted in adjustments of the questions. The questionnaires were face-to-face validated with prostate cancer patients and content validated by experts in urology. A detailed description of the study design, the procedure and development of the clinical record forms and the validation of the questionnaires has been published previously [23]. Two research nurses monitored the recruiting sites and all means for collecting data were standardized. In the questionnaire there are questions regarding QoL in general prior surgery that have been included in this study to describe the study population. Physical and psychological well being as well as self-assessed global quality of life were assessed on seven-point Likert type scale from 1 to 7 anchored by for example, no physical well-being and the best possible physical well-being. The questions were dichotomized according to previous studies by Steineck et al [29] with the lowest five of seven possible categories collapsed.

#### Physical activity measurement

The questionnaires included a question regarding the patient’s self-assessed level of physical activity: “How often have you been physically active for 30 min or more with for example riding a bike, walking, gymnastics or similar the last month?” The available options were; “Never”; “Sometimes” (one to two times a week); “Often” (three to four times a week); and “Daily or almost daily” (five to seven times a week). The highest two levels of self-assessed activity (>three to four times/week) corresponds to fulfilling the national and international criteria for recommended levels of regular physical activity, established to be associated with health benefits [30].

### Outcome measures

The primary outcome measure sick-leave was reported by the patients in the three-month postoperative questionnaire. The question read: “How long were you on sick-leave after the operation? The answering options were: “Not applicable, I was not on sick-leave”, “0 weeks”, “1-3 weeks”, “4-6 weeks”, “7-9 weeks”, “10-12 weeks”, “longer than 13 weeks (I am still on sick-leave)”.

In Sweden the legislation does not require doctor’s note, if sick-leave it is less than one week, thus “not applicable” and “0 weeks” were combined and used as a cut-off to define “no sick leave”.

In the questionnaire patients were asked to assess their time until full recovery, given as number of weeks. The question was: “How long time do you estimate that it took until you were back in your normal acitivy level after surgery?” Answering options were “Not applicable”, 0 weeks”, “1-3 weeks”, “4-6 weeks”, “7-9 weeks”, “10-12 weeks”, “longer than 13 weeks (I am still not back to normal activity level)”. This was then dichotomized into fully recovered prior to 4 weeks and not recovered at 13 weeks.

Regarding other outcome variables, the clinical record form at 6-12 weeks and the three-month postoperative questionnaire were used to assess hospital stay and adverse events. The adverse events were divided into five groups as follows; infection, cardiovascular, surgical, gastrointestinal and psychological events (Table 2). Sexual health and urinary incontinence were measured at one year, and were not the primary outcome of this study and are thus not reported in this study [25].

### Statistical analysis

As this analysis is a sub-study of the LAPPRO trial no power calculation was performed. The primary end-point of the LAPPRO trial was urinary leakage at 12 months, and with a significance level *p* = 0.05, 80 % power, two-sided test it was calculated to require 1400 robot-assisted laparoscopic radical prostatectomies and 700 open prostatectomies. Quality-of-life measurements were dichotomized as previously described [29]. Postoperative stay was compared between groups using Kruskal-Wallis test. Categorical data were compared between groups using χ^2^-tests, trends across groups were evaluated using Jonckheere-Terpstra’s test. Two sided *p*-values ≤0.05 were considered statistically significant. We also calculated relative risk adjusted for possible confounders using multivariable logistic regression models both with logit-link and log-link functions. Possible confounders: age, level of education, smoking, body mass index (BMI), American Society of Anaesthesiologists (ASA) classification, and alcohol consumption were all evaluated in univariable analyses against the primary outcome sick leave. Factors being significant at the 5 % level in univariable analyses were all included in the base multivariable model; other factors were entered one at the time in order to evaluate their effect on the association between level of physical activity and sick leave.

## Results

The demography is displayed in Table 1, with the patients being divided into the four groups, according to their self-assessed physical activity-level. The group answering “Never” was a minority, comprising of 102 patients (6.5 %). The patients in this group differed significantly from the others with a higher co-morbidity according to the American Society of Anaesthesiologists (ASA) classification and they reported smoking and drinking more often. To reduce the risk of including patients unable to exercise, these patients were excluded from the analyses regarding sick leave. Quality of life was higher in the patient group with a higher level of physical activity (Table 1) at base line. No other statistically significant differences were found at baseline between the three remainin groups of PA.

**Table 1:** Demography. Men operated with open or robot-assisted laparoscopic prostatectomy between 2008-2011 age ≤ 65 years old at 14 centers in Sweden. Divided into the four different categories of physical activity^a^

	Never	Sometimes	Often (3-4 times a week) (*n* = 466)	Daily or almost daily (5-7 times a week) (*n* = 403)	*p*-value	Missing
	(*n* = 102)	(1-2 times a week) (*n* = 598)				
Type of surgery					*p* = 0.271	2 (0.1 %)
Open	27 (26.5 %)	151 (25.3 %)	107 (23.0 %)	112 (27.8 %)		
Robot-assisted	75 (73.5 %)	446 (74.6 %)	358 (76.8 %)	291 (72.2 %)		
Preoperative characteristics						
Age, median	58 (37-64)	59 (39-64)	59 (39-64)	59 (39-64)	*p* = 0.330	2 (0.1 %)
Level of Education					0 = 0.001	0 (0 %)
University	35 (34.3 %)	222 (37.1 %)	222 (47.6 %)	187 (46.4 %)		
Vocational school	13 (12.7 %)	73 (12.2 %)	48 (10.3 %)	32 (7.9 %)		
Secondary school	29 (28.4 %)	210 (35.1 %)	144 (30.9 %)	129 (32 %)		
Elementary school	24 (23.5 %)	91 (15.2 %)	47 (10.1 %)	52 (12.9 %)		
Other	1 (1 %)	2 (0.3 %)	5 (1.1 %)	3 (0.7 %)		
BMI, median	27.1 (19-38)	26.3 (19-40)	26.2 (19-41)	25.7 (19-54)	*p* = 0.041	0 (0 %)
Smoking					*p* = 0.0005	13 (0.8 %)
Non-smoker	38 (37.3)	237 (39.6 %)	230 (49.4 %)	171 (42.4 %)		
Former smoker	42 (41.2 %)	278 (46.5 %)	200 (42.9 %)	204 (50.6 %)		
Current smoker	15 (14.7 %)	56 (9 %)	30 (6.4 %)	19 (4.7 %)		
Current snuff user	16 (15.7 %)	93 (15.6 %)	71 (15.2 %)	63 (15.6 %)	*p* = 0.996	11 (0.7 %)
High alcohol consumption^b^	3 (2.9 %)	6 (1 %)	2 (0.4 %)	4 (1 %)	*p* = 0.121	17 (1.1 %)
American Society of Anaesthesiology (ASA) classification					*p* = 0.760	41 (2.6 %)
I	67 (65.7 %)	421 (70.4 %)	336 (72.1 %)	295 (73.2 %)		
II	32 (31.4 %)	154 (25.8 %)	109 (23.4 %)	99 (24.6 %)		
III	2 (2.0 %)	3 (0.5 %)	6 (1.3 %)	1 (0.2 %)		
Gleason score					*p* = 0.562	105 (6.7 %)
< 7	54 (52.9 %)	314 (52.5 %)	245 (52.6 %)	198 (49.1 %)		
≥ 7	42 (41.2 %)	238 (39.8 %)	192 (41.2 %)	173 (42.9 %)		
Stroke	1 (1 %)	2 (0.3 %)	1 (0.2 %)	2 (0.5 %)	*p* = 0.774	10 (0.6 %)
Thromboembolic disease	1 (1 %)	8 (1.3 %)	6 (1.3 %)	5 (1.2 %)	*p* = 0.990	12 (0.8 %)
Neurologic disease	3 (2.9 %)	5 (0.8 %)	2 (0.4 %)	6 (1.5 %)	*p* = 0.245	11 (0.7 %)
Diabetes	4 (3.9 %)	28 (4.7 %)	20 (4.3 %)	17 (4.2 %)	*p* = 0.924	13 (0.8 %)
Hypertension	33 (32.4 %)	182 (30.4 %)	121 (26 %)	118 (29.3 %)	*p* = 0.269	11 (0.7 %)
Diagnosed depression	3 (2.9 %)	15 (2.5 %)	11 (2.4 %)	5 (1.2 %)	*p* = 0.363	16 (1.0 %)
Angina pectoris	0 (0 %)	9 (1.5 %)	7 (1.5 %)	3 (0.7 %)	*p* = 0.518	11 (0.7 %)
Long-term pain, unspecified	12 (11.8 %)	48 (8.0 %)	33 (7.1 %)	24 (6 %)	*p* = 0.472	15 (1 %)
Low or moderate subjective quality of life^c^	62 (60.8 %)	314 (52.8 %)	217 (46.6 %)	174 (43.3 %)	*p* = 0.004	4 (0.3 %)
Low or moderate physical well-being^c^	59 (58.4 %)	281 (47.2 %)	168 (36.1 %)	126 (31.5 %)	*p* < 0.0001	7 (0.4 %)
Low or moderate psychological well-being^c^	60 (59.4 %)	333 (56.0 %)	227 (48.7)	177 (44.1 %)	*p* = 0.003	6 (0.4 %)
Decreased general physical capacity^c^	67 (65.7 %)	264 (44.4 %)	164 (35.2 %)	126 (31.3 %)	*p* < 0.0001	4 (0.3 %%)
Self assessment of current health status (median with range in parenthesis)^d^	80 (30-100)	83 (10-100)	85 (15-100)	90 (30-100)	*p* < 0.0001	27 (1.7 %)

^a^Unless otherwise stated data is given as number with percentages or range in parenthesis. ^b^ Risk consumption of alcohol is defined as more than 15 glasses/week. ^c^The lowest five of seven possible categories. ^d^On a scale from 0-100. Categorical data were compared between groups using chi-square-tests, trends across groups were evaluated using Jonckheere-Terpstra’s test. Two sided *p*-values ≤0.05 were considered statistically significant

In Table 2 hospital stay, sick leave, and complications are displayed as well as current health status. A significantly larger proportion of patients (13 %) in the most active group reported a sick leave of shorter duration than one week. Most patients did not report full recovery within four weeks, but although statistically non-significant, it was slightly more common to be recovered within three months in the group with the highest level of self-assessed physical activity.

**Table 2:** Outcomes

	Sometimes (1-2 times a week) (*n* = 598)	Often (3-4 times a week) (*n* = 466)	Daily or almost daily (5-7 times a week) (*n* = 403)	*p*-value	Missing
Median length of post-op stay (days)	3 (1-18)	3 (2-15)	3 (1-8)	*p* = 0.561	130 (8.9 %)
Number of patients reporting no sick-leave or sick-leave less than one week	36 (6.3 %)	41 (9.2 %)	51 (13 %)	*p* < 0.001*	56 (3.8 %)
Feel fully recovered < 4 weeks postoperatively (%)	85 (14.9 %)	74 (16.9 %)	67 (17.5 %)	*p* = 0.214	56 (3.8 %)
Still not recovered at 3 months	76 (13.4 %)	53 (12.1 %)	42 (10.9 %)	*p* = 0.510	56 (3.8 %)
Any complication					
Infection^a^	118 (20.6 %)	93 (21.0 %)	73 (18.6 %)	*p* = 0.659	60 (4.1 %)
Cardiovascular^b^	28 (5.0 %)	22 (5.0 %)	21 (5.4 %)	*p* = 0.955	70 (4.8 %)
Surgical^c^	105 (18.5 %)	88 (20.0 %)	66 (16.8 %)	*p* = 0.493	64 (4.4.%)
Gastrointestinal^d^	72 (12.7 %)	56 (12.7 %)	47 (12.0 %)	*p* = 0.937	64 (4.4 %)
Psychological^e^	80 (14.2 %)	47 (10.8 %)	57 (14.7 %)	*p* = 0.182	78 (5.3 %)
Self assessment of current health status (median with range in parenthesis)	80 (5-100)	80 (19-100)	85 (19-100)	*p* = 0.02**	70 (4.7 %)

Postoperative stay, sick leave, evaluation of recovery and complications at 6-12 weeks postoperatively

Unless otherwise stated data are given as number with percentages or range in parenthesis. ^a^On a scale from 0-100. n.s. denotes not statistically significant. Postoperative stay was compared between groups using Kruskal-Wallis test. Categorical data were compared between groups using χ2-tests, trends across groups were evaluated using Jonckheere-Terpstra’s test. Two sided *p*-values ≤0.05 were considered statistically significant. *Jonckheere-Terpstra’s trend test. **Kruskall-Wallis test. ^a^Infection in the operating wound, pneumonia or urinary tract infection. Number of patients in the analysis: 1407 ^b^ Pulmonary embolism, hypertension, acute myocardial infarction, arrhythmia or other heart diseases, deep venous thrombosis, stroke. Number of patients in the analysis: 1397. ^c^Pain in the operating wound, pain in the lower abdomen, pain in the upper abdomen, bleeding from the operating wound, bleeding from the urinary tract, inguinal hernia. Number of patients in the analysis: 1403. ^d^Nausea, impaired appetite, loose or frequent stools, constipation. Number of patients in the analysis: 1403. ^e^Depressed mood, worry. Number of patients in the analysis: 1389

Higher age (mean 60.2 SD 3.5 vs. mean 57.9 SD 4.7, *p* < 0.0001) and higher educational level (521 patients with University degree reported 1 week or more compared to 760 patients with lower educational level, *p* < 0.0001) were both associated with shorter sick leave. However, when adjusting for these factors in a multivariate model (Table 3) the relationship between sick leave and level of physical activity remained, even when adding other potential confounders to the multivariate model one at a time.

**Table 3:** Unadjusted and adjusted analysis of the relationship between physical activity and sick leave

	Unadjusted OR (95 % CI)	*p*-value	Adjusted OR (95 % CI)^a^	*p*-value
Sometimes (1-2 times a week) versus Often (3-4 times a week)	0.66 (0.41-1.05)	*p* = 0.079	0.73 (0.45-1.20)	*p* = 0.215
Sometimes (1-2 times a week) versus Daily or almost daily (5-7 times a week)	0.45 (0.29-0.70)	*p* = 0.0004	0.49 (0.30-0.78)	*p* = 0.003
Often (3-4 times a week) versus Daily or almost daily (5-7 times a week)	0.68 (0.44-1.06)	*p* = 0.083	0.66 (0.42-1.05)	*p* = 0.080

^a^Adjusted for possible confounders using multivariable logistic regression models both with logit-link and log-link functions. Factors being significant at the five percent level in univariable analyses were all included in the base multivariable model; other factors were entered one at the time in order to evaluate their effect on the association between level of physical activity and sick leave. Adjusted for educational level, age, ASA-classification, alcohol consumption, smoking, BMI and surgical technique

## Discussion

The main finding of the present study is that a higher level of self-assessed regular physical activity preoperatively was associated with a reduction in sick-leave after radical prostatectomy. Furthermore, we found that the self-assessed current health status was higher in the more physically active group, three months after surgery.

The finding that a higher level of physical activity preoperatively may reduce sick leave have important clinical implications. First of all, the quality of life for many patients still working, may be increased with an early return to work. Secondly, as many treatments and procedures aim to shorten hospital stay and reduce sick leave, increased levels of physical activity may reduce societal costs. While physical activity is known to have health benefits in patients with cancer [31], this study adds to the present knowledge by showing that also the postoperative recovery may be improved by having a higher level of physical activity before surgery.

The patients in our study in general had a shorter sick leave than what has previously been reported from in a similar setting [21], and this was especially true for the open prostatectomy group. This may be due to the standardized period of short sick leave, enabling patients to be on paid sick leave for a more exact time, as warranted, less influenced by any decision made by the doctor.

In this study no clear differences were found between the different groups of physical activity regarding postoperative complications or hospital stay, possibly due to the short hospital stay in itself.

In addition, the finding that the quality of life is associated with the level of preoperative physical activity is important as it is confirming earlier studies have shown that quality of life in general is improved in cancer patients with a higher level of physical activity [32, 33].

The strengths of this study include the prospective design and a large number of included patients. Furthermore, all patients we analysed answered a preoperative questionnaire, giving a baseline status. A limitation may be that we used a question to assess physical activity, that has not been validated in relation to long-term morbidity, such as the Saltin-Grimby scale has [34]. However, our questions uses a longer time frame (one month) than several other scales, which is good in this setting of patients with cancer waiting for surgery, as it may more clearly depict the patient’s everyday habits, but this remains to be shown. One may speculate some occupations imply less need for a formal sick leave than other, and that in the same occupations the men are able to, or have the habit to, enrol in a high level of physical activity. We have no data to support this speculation. However, it is difficult to speculate upon to what extent this has affected our results. The two higher levels of physical activity on the scale correspond to national guidelines indicating their value in the clinical setting [35].

Another limitation may be that more than 70 % were operated with one technique (robot-assisted laparoscopic radical prostatectomy), however, surgical technique was included in the adjusted analysis and did not affect results. It is also possible that pre- and postoperative care differed between the different hospitals, however, due to the fact that center also was related to type of procedure this is difficult to fully cover in the adjusted statistical analysis. Another limitation is that the patients did not address whether their job was physically demanding or more office work prior to surgery, but to some extent this is made up for by the use of educational level as this often correlates well.

A future challenge is how to achieve a higher level of physical activity preoperatively for any patient group. Methods to increase the level of physical activity in patients have been introduced in recent years. The Swedish model of physical activity on prescription has been shown to increase the level of physical activity [36], and to positively affect metabolic risk factors [37]. The National Board of Health and Welfare now recommend physical activity for all insufficiently physically active patients in Sweden (www.socialstyrelsen.se) [35]. The population of prostatectomy patients may be a target population for recommendations on similar physical activity interventions.

## Conclusions

We found that a higher level of physical activity preoperatively was associated with a reduced sick leave for patients after radical prostatectomy in a Swedish setting. Further studies are required, but it is possible that a recommendation on individualised physical activity, prior to surgery, could be included in the preoperative programme aimed for patients being planned for prostatectomy.

## Abbreviations

ASA-classification, American Society of Anaesthesiologists classification; BMI, body mass index; LAPPRO, LAParoscopic prostatectomy robot open.
